# Strategic Improvement for Quality and Satisfaction of Hospital Information Systems

**DOI:** 10.1155/2018/3689618

**Published:** 2018-09-12

**Authors:** Kuang-Ming Kuo, Chung-Feng Liu, Paul C. Talley, Su-Ya Pan

**Affiliations:** ^1^Department of Healthcare Administration, I-Shou University, No. 8, Yida Rd., Yanchao District, Kaohsiung City 82445, Taiwan; ^2^Department of Information Management, Chia-Nan University of Pharmacy and Science, No. 60, Erh-Jen Rd., Sec. 1, Jen-Te District, Tainan City 71710, Taiwan; ^3^Department of Applied English, I-Shou University, No. 1, Sec. 1, Syuecheng Rd., Dashu District, Kaohsiung City 84001, Taiwan; ^4^Department of Medical Record Management, E-Da Hospital, No. 1, Yida Rd., Yanchao District, Kaohsiung City 82445, Taiwan

## Abstract

The purpose of our study aimed to identify attributes capable of improving physicians' satisfaction levels with the use of a hospital information system (HIS). A model inclusive of system quality, information quality, and service quality related to an HIS is used to form antecedents of user satisfaction. Survey methodology was used to collect an attributive set representing the system quality, information quality, and service quality made available from 150 physicians at a large health-care system in southern Taiwan. Responses were segmented into low and high satisfaction and analyzed with partial least squares and importance-performance analysis. The results reveal that system quality, information quality, and service quality may be used to significantly predict physicians' satisfaction. Two system quality attributes (reliability and response time) were identified as the highest priorities for intervention by low- and high-satisfaction users. Low-satisfaction users further expect improvement of the HIS service quality to take place. The subject health-care system should produce coping interventions for those high priorities to enhance the satisfaction of physicians.

## 1. Introduction

With the rising cost pressure and the increasing demand for improved health-care quality, the larger number of health-care facilities has forced the introduction of health information technologies (HIT) to resolve myriad problems that have arisen. By leveraging HIT with an integration of hospital functions and processes, hospital information systems (HIS) are frequently adopted among most health-care facilities to meet administrative requisites. A hospital's goal of adopting HIS is expected to potentially fulfill the strategic goal of improving overall patient-care quality and reducing costs [1, 2]. Furthermore, HIS usually requires heavy capital investments with respect to both staff and information systems processing [1]. However, literature [3] from the information systems (IS) field has clearly pointed out that IS cannot improve organizational performance unless it is fully utilized. The question invariably is how to go about increasing HIS use which is an important issue for health-care facilities choosing HIS. The adoptive users of HIS, who are best seen as an integral part of an HIS [1], therefore play a pivotal role on whether or not an HIS can ever achieve the expected goals of improving overall patient-care quality and reducing costs.

Prior evidence has found that user satisfaction has a direct association with IS usage intentions [4]. Earlier studies [5] also found that the users' level of satisfaction was positively related with their intention to continue the use of an IS. It is therefore entirely reasonable to assume that if users are more satisfied with HIS, the greater the likelihood there will be for them to integrate use of HIS for future patient care. Hence, it is strategically crucial for administrative managers to comprehend and to identify the primary HIS attributes perceived by users as becoming important, and to scrutinize how users perceive the given performance of those attributes once adoption is implemented.

The purpose of our study is therefore to determine the most important attributes of HIS related to perceived physicians' satisfaction since they are the primary users of HIS, and they are heavily reliant on HIS to affect efficient patient diagnosis and meaningful treatment. By identifying the priorities of improvements for those given attributes, hospital administrators can plan and then take appropriate steps for intervention to occur that will improve HIS in order to fulfill physicians' clinical requirements for providing better health-care quality.

### 1.1. Hospital Information Systems

According to the literature [1], a hospital information system (HIS) is defined as “*the socio-technical subsystem of a hospital, which comprises all information processing as well as the associated human or technical actors in their respective processing roles”* (p.30). HIS can also be defined as an integrated information system that supports the various information requirements of clinical services and hospital management [6]. Therefore, HIS ranges from a simple system, such as transaction processing systems, all the way up to complicated systems, such as clinical decision support systems [7]. More specifically, HIS may include various and diverse types of health-care IS [6, 7], such as clinical information systems, radiology information systems, laboratory information systems, pharmacy information systems, clinical decision support systems, nursing documentation systems, computerized physician order entry, patient-centered information systems, or administrative information systems, etc. No matter what type of HIS is in use, the primary aim of HIS is to contribute to better quality and efficient patient care [1]. In the beginning, HIS was aimed at supporting the regular fiscal operations and administrative aspects of a given hospital [2]. Nowadays, health-care facilities leverage their HIS mainly to focus on providing better possibilities for patient care and its management to take place, in addition to optimizing operational standards [2]. And, HIS has become an indispensable part of the diagnostic and treatment processes of health-care professionals [7].

Among those different types of HIS, computerized physician order entry (CPOE), just one type of HIS primarily used by physicians, is gaining popularity among health-care facilities. Via CPOE, physicians can acquire clinical decision support such as drug-drug interaction and drug-allergy checks during the relative diagnosis and treatment of patients [7]. Without these decision-support capabilities, patient safety can be compromised if physicians misunderstand or neglect such important sources of patient information. The importance of HIS for improving health-care quality is thus most evident. Therefore, the extent to which HIS fulfills its role and supports health-care services cannot be overemphasized.

Many studies have been conducted regarding HIS, and these studies have lain special focus on how to encourage HIS adoption [8]. The findings surely have advanced our knowledge toward what factors motivate health-care professionals for adopting an HIS. However, if the use of HIS is mandatory, the method of how to increase their usage is an additionally important issue that should be focused upon. Furthermore, since there are many types of HIS, with each possessing differing characteristics and users, it may be better to focus on specific HIS and possible users to better understand those determinants that can improve HIS use by its end users.

### 1.2. Information Systems Success Model

**Figure 1:**
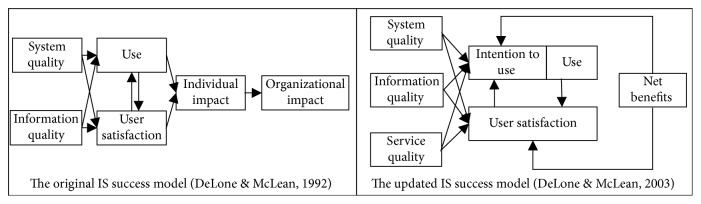
Information systems success model.

To assess the success of an IS, DeLone and McLean [9] introduced a taxonomy comprising six measures of IS success by reviewing 180 related studies. They proposed an IS success classification framework inclusive of system quality, information quality, use, user satisfaction, individual impact, and organizational impact. These dimensions are interrelated both causally and temporally. As shown in Figure 1, system quality and information quality together impact both use and user satisfaction which, in their turns, are precursors of individual impact. Finally, individual impact affects organizational impact. DeLone and McLean, however, do not empirically assess their model but suggest further refinement and corroboration of the model they proposed [9]. Numerous studies have extensively tested this model [10–15]. According to the previous literature, DeLone and McLean [16] included service quality as a new dimension to update their originally proposed model for better measuring IS success, further consolidating all the “impact” measures into one construct labeled as “net benefits” (Figure 1).

Prior literature has adopted the information systems success model (ISSM) for assessing the success of an IS including those used in the health-care field [11–13, 17]. The subjects of these studies included health-care professionals such as nursing staff or a mix of physicians, nurses, and other health-care professionals. To our knowledge, studies that mainly focused on the exclusive perceptions of physicians are scarce. These studies generally support the ISSM [9, 16]. However, in a meta-analysis of ISSM [14], the relationship between service quality and user satisfaction was not supported. One of the potential reasons that Petter and McLean [14] proposed for this is the possible consideration of population choice. It is therefore necessary to focus on a specific group of HIS users in order to gain a deeper knowledge of those associations observable among the constructs proposed by ISSM.

### 1.3. Importance-Performance Analysis

**Figure 2:**
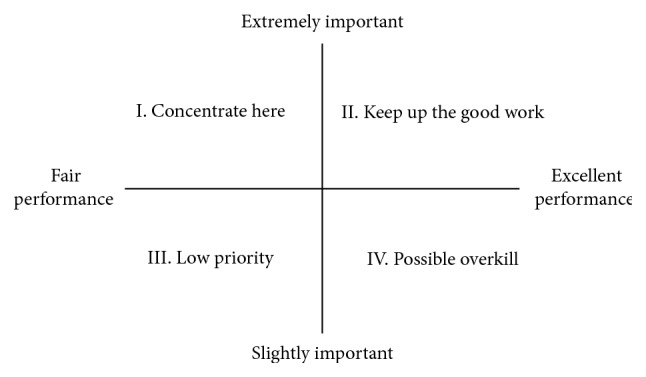
Importance-performance grid.

Stemming from the marketing discipline, the importance-performance analysis (IPA) technique has often been utilized for strategy formulation, leading to service improvement in different fields [12, 18, 19]. The IPA analyzes quality attributes on the basis of performance and importance dimensions. These two dimensions are then integrated into a matrix with four quadrants: (1) “Concentrate here,” (2) “Keep up the good work,” (3) “Low priority,” and (4) “Possible overkill.” Respondents are asked to indicate the relative attribute's importance and performance merits. And, the mean values of importance and performance ratings are often used to divide the quadrants. The ratings of importance and performance are then plotted on the importance-performance grid (Figure 2). For example, if customers feel an attribute is important but its performance is low, then this attribute falls on the “I. Concentrate here” quadrant. Organizations would thus be best served to pay immediate attention to improve this desired attribute.

The original IPA is easy to implement for discovering strategies, leading to improved organizational services quality. The IPA, however, may suffer from the problem of ceiling effects which may inflate the importance ratings of most attributes [18] since the respondents have to rate importance and performance simultaneously [19]. In order to resolve this issue, some researchers adopted statistical methodologies such as correlation analysis [20], linear regression [21], and partial least squares [22] to derive relative importance.

In the health-care field, Cohen et al. [12] used IPA to assess important HIS attributes from a nursing perspective. They adopted partial least squares to derive the most important HIS attributes as high priorities for purposes of intervention. The findings of Cohen et al. [12] have absolutely added to the knowledge of the applicability of IPA, especially in health-care context. We, however, consider that if the perspectives of physicians, another key HIS user group, responsible for the diagnosis and treatment of patients should also be investigated, a more holistic view of the applicability of IPA in health-care context may then be acquired. Furthermore, healthcare facilities can take suitable interventions to improve HIS, thus achieving better patient care.

### 1.4. Conceptual Background

The ISSM, proposed by DeLone and McLean [9, 16], provides a useful framework for any understanding of the influence IS attributes have on user satisfaction. ISSM articulates that system quality, information quality, and service quality are important dimensions for users to evaluate the success of IS [9, 16]. The ISSM has been applied to various fields for IS evaluation, and it has been found applicable for health-care professionals [11–13, 17], including physicians [11].

**Figure 3:**
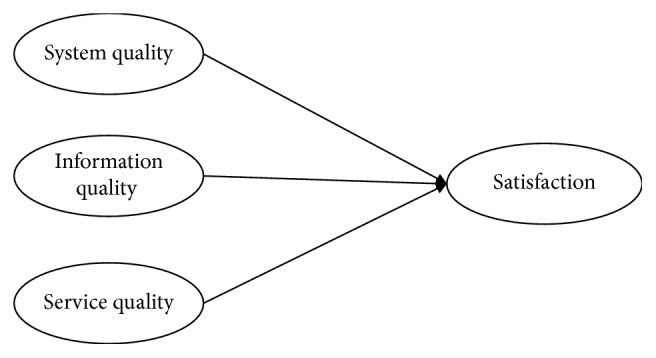
Research framework.

To determine the most important attributes of HIS related to a physician's satisfaction, we proposed a research framework based on the ISSM. As shown in Figure 3, the framework demonstrates that system quality of, information quality of, and service quality of an HIS are all expected to influence physicians' satisfaction related to HIS. And, system quality, information quality, and service quality are composed of their respective attributes.

#### 1.4.1. User Satisfaction

User satisfaction, referring to the affective response or attitude of physician users toward HIS, is considered an imperative indication of IS success [9, 16]. Lower levels of satisfaction, implying HIS may not meet the demands of physician users, can result in low or nonuse of HIS by physician users. In a health-care context, such inefficiencies in HIS usage may disturb the delivery of care and increase the possibility of new errors that may certainly jeopardize the quality and safety of patient health care [23]. On the other hand, an HIS that physician users are satisfied with can improve decision-making quality, performance, productivity, and effectiveness of patient-care tasks as the evidence illustrated [13, 17, 24].

According to the IS success model [9, 16] and other evidence [13, 17, 25], HIS attributes including system quality, information quality, and service quality may prove to be important antecedents of user satisfaction.

#### 1.4.2. System Quality

System quality measures desired technical characteristics such as the reliability, response time, and functionality of an HIS [9, 16]. These attributes have been confirmed to be important predictors for health-care professionals to use HIT applications [13, 17, 26, 27]. An unreliable HIS may lead to several unintended consequences such as additional workload and negative emotional states for the health-care professionals involved [28, 29].

#### 1.4.3. Information Quality

Information quality relates to the characteristics of the information output derived, such as sufficient detail, easy-to-read perception, and the completeness offered by an HIS [9, 16]. Previous studies have proved that health-care professionals can improve their ability to make better diagnoses, treatment plans, and patient-care provisions by acquiring patient information with the abovementioned attributes [13, 17]. By perceiving these tangible benefits, they can also have a better view of HIT [13]. Based on this finding, information quality should be seen as an important determinant of physician users' satisfaction on HIS.

#### 1.4.4. Service Quality

Service quality concentrates on the level of support, such as availability, responsiveness, and training opportunities, of physician users by the IS department [9, 16]. The marketing literature [30] has found strong empirical notice that improved service quality can lead to consumers' behavioral intention. In the same vein, treating physician users as internal customers and providing them with appropriate support and training should attract them to use an HIS even when and if they encounter perceived problems. Previous evidence also demonstrates the association between service quality and HIT implementation and adoption [31].

Evidence [14] found that ISSM has been less validated as a whole in the form of a single study. Furthermore, the use of an HIS in the subject hospitals is mandatory; our study, therefore, does not incorporate the use construct but only constructs that are inclusive of system quality, information quality, service quality, and satisfaction as found in the proposed model. According to a meta-analysis of ISSM [14], system quality, information quality, and service all have a significant relationship with user satisfaction. The strength of these relationships is ranked as follows: system quality, information quality, and service quality. Our study adapted the operational definitions of previous ISSM [9, 16] and altered them for use in the HIS context as provided. The constructs used in our study and in their HIS-specific definition are demonstrated in Table 1.

## 2. Materials and Methods

### 2.1. Measures

Based on Churchill's [32] suggestion for developing questionnaires, we derived survey items from prior validated surveys to establish an initial pool of survey items for each of the given constructs. An expert panel, including two experienced attending physicians employed by the subject health-care system and one health-care information management scholar, assessed the face and content validity of those proposed items. Suggestions offered by the panel were used to modify some ambiguous words in order to minimize plausible confusion during the survey's subsequent administration.

The questionnaire utilized in our study consisted of two parts. The first part elicits physicians' demographic information and the second section is designed to ascertain physicians' perceptions regarding the four constructs to be explored (i.e., system quality, information quality, service quality, and satisfaction). Our investigated constructs were assessed by adopting existing instruments [10–12, 15, 33], and a seven-point Likert scale was used to measure the items (e.g., one for “strongly disagree,” four for “neither agree nor disagree,” and seven for “strongly agree”). Regarding the detailed sources of survey items, system quality was measured using three items based on Balaban et al. [10] and Xu et al. [33]. Information quality was measured using three items adapted from Bossen et al. [11] and Xu et al. [33]. Service quality was measured using four items in accordance with Balaban et al. [10] and Wang [15]. Satisfaction was measured using five items taken from Cohen et al. [12].

Following the suggestion of Straub [34], we conducted a pilot test to establish the scales via a sampling of 10 attending and resident physicians employed at the subject health-care system. Slight modifications of wordings were made to the given items, and Table 2 lists the revised final scale (Table 2) justified for further validation.

### 2.2. Sampling

A cross-sectional survey was conducted to obtain data from the physicians of a large health-care system located in southern Taiwan. The subject health-care system comprises three different scales of hospitals, which have nearly 1,600 beds and which employ about 190 full-time physicians. The study chose the subject health-care system for two primary reasons: (1) The subject hospitals provide nearly all necessary/comprehensive medical services to their respective communities, which attracts an average of 7,000 outpatients each day, and (2) the subject health-care system has adopted the same HIS, with slightly different functionalities according to the requirements of respective hospital, since 2004. Since patients may need to be transferred among these three hospitals, an effective HIS is critical for exchanging patient information among physicians. With a growing demand from both administration and health-care perspectives, the functionalities of a current HIS may not conform to the demands of physicians, who are the primary users.

Ethical approval from the institutional review board of the respective large Taiwanese hospital was acquired prior to the commencement of the study (IRB#: EMRP-105-128). Prior to the distribution of the questionnaires, the researchers successfully contacted the relevant medical service departments to ensure their collaboration. We then designated a coordinator for those units of the medical service departments which were involved and who were both voluntarily and willing to assist with the dispatching and collection of the questionnaires per se. Altogether, 180 questionnaires were dispatched to those units. Physicians were recruited by our coordinator to anonymously and voluntarily participate in our survey. In total, 150 useful responses were returned, resulting in a response rate of 78.95% (150/180), for the purpose of later analysis.

### 2.3. Statistical Analysis

In addition to descriptive statistics, several quantitative procedures were involved for importance-performance assessment in our study. First, exploratory factor analysis with oblique rotation was employed to prove construct validity before conducting IPA as suggested by literature [18]. Next, partial least squares (PLS) was used to test the measurement model and structural model. The measurement model articulates the associations between the latent variables and the measured variables, whereas the structural model articulates the associations between the exogenous and endogenous latent variables [35]. Importance scores and performance scores, of both construct level and item level, were then derived from PLS results, respectively [22, 36].

In order to obtain different perspectives from a traditional utilization of IPA, a median of factor means based on original 7-point scales of satisfaction (4.2) was used to split the data into high-satisfaction and low-satisfaction groups. An independent sample *t*-test was conducted to compare satisfaction mean scores between low- and high-satisfaction groups of respondents, and a significant difference was noted between low satisfaction (*M* = 2.98, SD = 0.85) and high satisfaction (*M* = 4.99, SD = 0.69); *t* (130.74) = −15.65, *p* < 0.001. IPA was then employed to compare the perceptions of HIS attributes by physician users between high-satisfaction and low-satisfaction groups. The above statistical analysis were conducted by using R software [37] with the matrixpls package [38].

## 3. Results

### 3.1. Descriptive Statistics

Of the 150 useable responses, most respondents were male (79.33%), close to the national average gender ration of physicians [39]. Nearly 82% of the respondents were aged between 30 and 49 years . All respondents have at least a college or university education, respective Taiwanese regulations respective to becoming physicians. General surgery accounts for the primary group of respondents (38.46%) and 68% of respondents are attending physicians. And, 78% of the respondents have more than 4 years of working experience in this particular health-care system. All physicians are mandated to use HIS when caring for their patient workload, showing that the respondents are suitable for the purposes of our study. Furthermore, high-satisfaction users seem to be senior (*M*: 2.27 vs. 2.13), and have more work experience (*M*: 3.69 vs. 3.64) and HIS usage frequencies (*M*: 3.57 vs. 3.52) than low-satisfaction users. Detailed demographic information of the respondents is shown in Table 3.

### 3.2. Exploratory Factor Analysis

Table 4 shows the reliability and validity results of the constructs used in this study. The Cronbach's *α* values range from 0.83 to 0.95, indicating sufficient reliability [40]. The Kaiser-Meyer-Olkin (KMO) measure verifies the sampling adequacy with KMO = 0.94 [40]. Bartlett's test of sphericity, *χ*^2^ (105) = 2120.27, *p* < 0.001, demonstrating correlations of items, are sufficient for principal components analysis [40]. With oblique rotation, four factors with eigenvalues of at least one were extracted. Convergent validity can be confirmed if the items load significantly on their respective factors, while discriminant validity can be verified if each of the items load higher on its posited factors than on other factors [40]. Table 4 demonstrates that all items have loadings of >0.45 on their posited factors and load higher on the posited factors than on others. Reliability and construct validity are thus determined to be adequate in our study.

### 3.3. Partial Least Squares Modeling Results

In accordance with the suggested procedures for conducting PLS [35], we adopted a two-stage process, measurement model, and structural model, for assessing the PLS model. Furthermore, based on suggestions made in prior IPA literature [12, 36], HIS attribute and satisfaction scores were first rescaled from 0 to 100 by using the equation proposed by Anderson and Fornell [41] before conducting PLS analysis. Furthermore, in order to compare the perceptions of differing satisfaction level, the following data were analyzed with full, high-satisfaction, and low-satisfaction datasets, respectively.

#### 3.3.1. Measurement Model

Regarding the evaluation of measurement model, reliability, convergent validity, and discriminant validity are often used to assess the measurement model of PLS per the suggested procedures by PLS guiding literature [35, 42]. We used composite reliability (CR) to evaluate the reliability [35, 42]. As shown in Table 5, the CR of four constructs in our study was larger than the suggested criterion of 0.7 [35]. As for convergent validity, the average variance extracted (AVE) of the constructs in our proposed model was larger than the threshold of 0.5 [42]. Furthermore, the matrix of inter-construct correlations (Table 6) demonstrated that the square root of AVE for each construct was higher than the association of the specific construct with any other constructs in our proposed model, thus revealing sufficient discriminant validity [42]. From the results, our study showed sufficient reliability and validity for the constructs to be investigated.

#### 3.3.2. Structural Model

**Figure 4:**
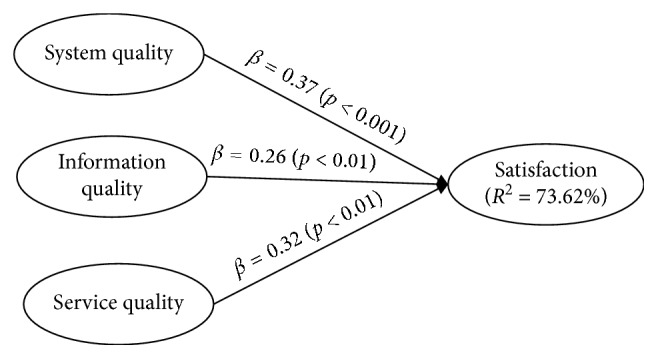
Structural model results.

We evaluated the results of the structural model by assessing the size and significance of path coefficients (*β*), coefficient of determination (*R*^2^), the strength of each predictor variable in explaining endogenous variables (*f*^2^), predictive relevance (*Q*^2^), and the relative impact of the predictive relevance (*q*^2^) as per the suggestions of PLS literature [35]. First of all, we adopted a bootstrapping procedure to assess the structural model for testing the significance of each path coefficient.  Figure 4 shows the results of structural model with path coefficient and probability values (*p* values). For the full-sample group, system quality predicted physicians' satisfaction on HIS significantly and positively (*β* = 0.37, *p* < 0.001), with a 95% bias-corrected and accelerated bootstrap confidence interval (BCa CI) of [0.11, 0.51]. Furthermore, both information quality (*β* = 0.26, *p* < 0.001, 95%) with a BCa CI of [0.08, 0.40] and service quality (*β* = 0.32, *p* < 0.001) with a 95% BCa CI of [0.17, 0.66] also predicted satisfaction significantly and in an expected direction. In addition to the link between information quality and satisfaction in the low-satisfaction group and the link between service quality and satisfaction in the high-satisfaction group, all other paths between system quality→satisfaction, information quality→satisfaction, and service quality→satisfaction reveal significant and positive relationships.

We then assessed effect sizes including *R*^2^, *f*^2^, *Q*^2^, and *q*^2^. For the coefficient of determination (*R*^2^), the model explains about 74% of the determined variance in the physician users' satisfaction regarding HIS usage. Furthermore, system quality, information quality, and service quality have *f*^2^ effect sizes of 0.21, 0.12, and 0.14 for explaining satisfaction, respectively. These figures represent medium-large, small-medium, and small-medium effect sizes for system quality, information quality, and service quality, respectively [43]. As for predictive relevance, the *Q*^2^ value of satisfaction (=0.53) is higher than zero, revealing that our model possesses sufficient predictive relevance for satisfaction construct [35]. The respective *q*^2^ effect sizes for system quality, information quality, and service quality are 0.04, 0.10, and 0.08, indicating small to medium relative predictive relevance [43].  Table 7 depicts the summary of path coefficients and 95% bias-corrected and accelerated bootstrap confidence intervals for three groups of data.

### 3.4. Performance Indices and Prioritization of Improvement Areas

Following the suggestions of IPA literature [12, 18], PLS path coefficients were used as construct-level importance scores, whereas item-level importance scores were derived by multiplying each HIS attribute's outer weights with relevant PLS path coefficients. Regarding construct level, performance scores were estimated as a weighted average of the attributes items, rescaled from the original 7-point scale into 0-to-100 point [41], whereas the item-level performance scores were simply calculated as the mean of rescaled scores of HIS attributes [36]. Crosshairs, using the mean values of importance and performance scores, were calculated to separate the latent variables and HIS attributes into four quadrants, namely, “I.Concentrate here,” “II. Keep up the good work,” “III. Low priority,” and “IV. Possible overkill.”

#### 3.4.1. Construct-Level IPA Results

**Figure 5:**
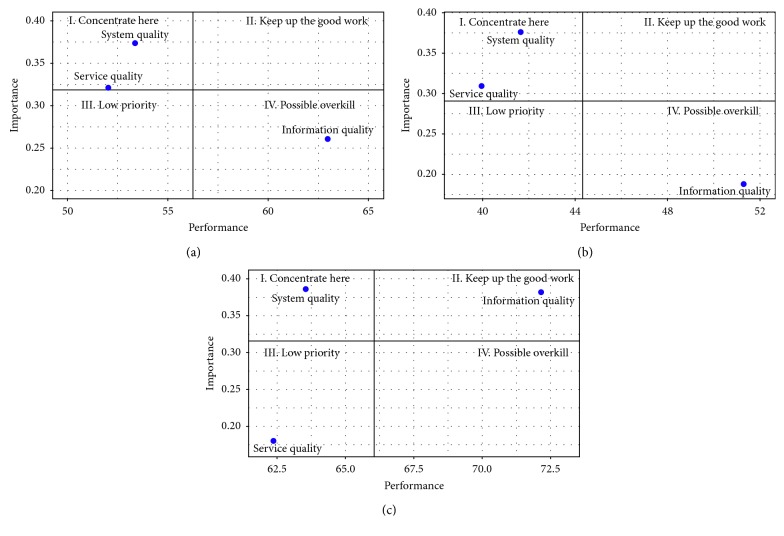
Construct-level IPA results. (a) Full sample. (b) Low satisfaction. (c) High satisfaction.

By first looking at construct-level IPA results, Table 8 clearly demonstrates that the performance of system quality and service quality for all three groups is below the mean performance index. Such results indicate that the system quality and service quality of an HIS have evident improvement potential for the subject health-care system. On the other hand, the information quality of an HIS is higher than the mean performance index for all three groups of data. Data from Table 8 were then transferred into the IPA grid representation. Figures 5(a)–5(c) depict the results of the IPA grids for the full-sample, low-satisfaction, and high-satisfaction data groups, respectively.

As shown in Figures 5(a) and 5(b), the full-sample and low-satisfaction groups of data have the same pattern of IPA grids. System quality and service quality were identified in the “Concentrate here” quadrant, whereas information quality was identified in the “Possible overkill” quadrant. Furthermore, Figures 5(b) and 5(c) show that low- and high-satisfaction group of respondents only had similar perceptions toward system quality, which was identified in the “Concentrate here” quadrant. Information quality was identified in the “Possible overkill” and “Keep up the good work” quadrants by low-satisfaction and high-satisfaction groups, respectively. Finally, service quality was identified in the “Concentrate here” and “Low priority” quadrants by low- and high-satisfaction groups, respectively. In order to identify those areas for interventions that are capable of improving overall system quality, information quality, and service quality of an HIS, the next step is to interpret the impact of all attributes for these three factors.

#### 3.4.2. Attribute-Level IPA Results

**Figure 6:**
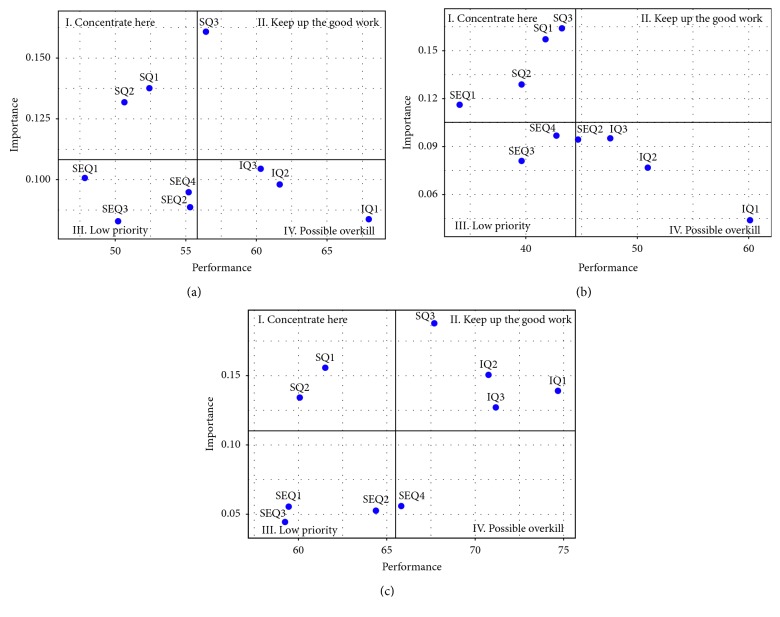
Item-level IPA results. (a) Full sample. (b) Low satisfaction. (c) High satisfaction.

Figures 6(a)–6(c) show the IPA grids of system quality, information quality, and service quality of an HIS from an attribute level for full-sample, low-satisfaction, and high-satisfaction groups of data, respectively. For the full-sample group, two system quality attributes, namely, SQ1 and SQ2, fall into the “Concentrate here” quadrant and one system quality attribute, namely, SQ3, falls into the “Keep up the good work” quadrant. The performance of SQ1 and SQ2 should therefore be improved, given their importance to engendering or maintaining user satisfaction. Furthermore, all three attributes of information quality fall into the “Possible overkill” quadrant, while all four attributes of service quality fall into the “Low priority” quadrant. It may indicate that the health-care system should consider reducing the organizational resources devoted to HIS information quality and service quality since physicians regard these two qualities as low priority or “Possible overkill” ones for improving HIS satisfaction. However, information quality and service quality are still considered to be critical determinants for HIS satisfaction, which was confirmed by means of the PLS analysis.

For low-satisfaction data, all three system quality attributes fall into the “Concentrate here” quadrant, while all three information quality attributes fall into the “Possible overkill” quadrant as with the full sample. Regarding service quality, two attributes, namely, SEQ3 and SEQ4, fall into the “Low priority” quadrant, while SEQ1 and SEQ2 fall into the “Concentrate here” and “Possible overkill” quadrant, respectively.

As for high-satisfaction data, two system quality attributes, namely, SQ1 and SQ2, were identified in the “Concentrate here” quadrant, while SQ3 was identified in the “Keep up the good work” quadrant. Regarding service quality, attributes including SEQ1, SEQ2, and SEQ3 fall into the “Low priority” quadrant, while SEQ4 falls into the “Possible overkill” quadrant. Item-level importance and performance scores are shown in Table 9.

#### 3.4.3. Comparing Construct-Level and Item-Level IPA Results

Compared with the results of the construct-level IPA, one interesting point may be noted. The identified quadrant of services quality factor (“Concentrate here”) in Figure 5(a) is not the same as that of the identified quadrant, namely (“Low priority”), of service quality for the full sample in Figure 5(a). If we look further at the IPA grids of Figure 6(b) for the low-satisfaction group, we can find that one attribute of service quality, namely, SEQ1, did fall into the “Concentrate here” quadrant, while two other attributes of service quality (i.e., SEQ3 and SEQ4) fall into “Low priority” quadrant and one attribute, namely, SEQ2, falls into the “Possible overkill” quadrant. SEQ1 is clearly an important priority for intervention to enhance physician-user satisfaction according to such findings. It may imply that physicians concern whether services provided by the IT department are sufficient; however, physicians can figure out how to intuitively use common functions of their own HIS by themselves.

Another interesting point should be noted regarding the differing perspectives on information quality by low-satisfaction and high-satisfaction groups. For low-satisfaction users, information quality was identified in the “Possible overkill” quadrant, while information quality was identified in the “Keep up the good work” quadrant by high-satisfaction users. Based on the demographic information, high-satisfaction users tend to have higher experience levels.

## 4. Discussion

Based on the results, system quality, information quality, and service quality significantly predict the satisfaction level of users of an HIS. The importance of and performance of HIS attributes can be derived from those results.

The construct-level IPA results shown above suggest that special attention should be devoted to system quality factors, regardless of differing satisfaction status toward the HIS by physicians. Respective physicians usually need to make critical and near-immediate decisions about patient treatment plans, so an unreliable or unresponsive HIS is certainly unacceptable [11]. Furthermore, integration of various functionalities such as diagnosing, medication, and lab-test ordering is also essential for increasing system quality, leading to physician satisfaction. Therefore, without regular, positive experiences with an HIS and its operational reliability, response time, and sufficient functionalities, the availability of an HIS will still be incapable of satisfying physician users.

As for information quality, the health-care system is clearly performing well on this factor despite the considerations of low- and high-satisfaction users having differing attachments to such levels of importance. A plausible reason for such an incongruence may be that high-satisfaction users have more work and HIS usage experience than low-satisfaction users since they have greater relative understanding of how to access required information from an HIS. Since the adoption of an HIS in early 2004, the general health-care system has undergone numerous developments and modifications of HIS implementation. New information requirements of physicians have been proposed and implemented in HIS gradually, albeit junior physicians may not well understand all of those functionalities which provide differing patient information as required. They may instead consider those functionalities to be unnecessary and even that the health-care system places too many resources toward improvement of HIS information quality.

Furthermore, service quality is another important factor that cannot be ignored for low-satisfaction users. Despite high-satisfaction users regarding service quality factors to be of low importance, they still consider the health-care system to perform unsatisfactorily within this aspect. The health-care system should not overlook the implication of this finding. Low-satisfaction users tend to be less experienced, according to the demographic information, with HIS than high-satisfaction users; therefore, they may require more assistance with HIS usage. This concern may explain why service quality factor was identified in the “Concentrate here” quadrant by low-satisfaction physicians.

Based on item-level IPA results, the “Concentrate here” quadrant captured two system quality attributes, namely, SQ1 and SQ2, for both low- and high-satisfaction HIS users. These findings suggest that the subject health-care system should direct its special attention on the reliability (SQ1) and response time (SQ2) aspects of an HIS. Besides, SQ3 was identified in the “Concentrate here” and “Keep up the good work” quadrant by low-satisfaction and high-satisfaction physicians, respectively. A possible reason for such a differing perspective may be due to their differing HIS usage experiences, as mentioned in the previous section. In addition to having more experiences with HIS, high-satisfaction physicians may involve with the continuous development of HIS functions. They are therefore more knowledgeable about the available functions provided by HIS than low-satisfaction physicians.

The same rationale can be used to explain the differing perspectives of the identified quadrant of both information quality and service quality attributes by low- and high-satisfaction physicians. Regarding information quality attributes, IQ1, IQ2, and IQ3 fall into the “Possible overkill” quadrant for low-satisfaction physicians, while IQ1, IQ2, and IQ3 fall into the “Keep up the good work” quadrant for high-satisfaction physicians. With continued HIS development, increasing and differing angles of patient information can be used by physicians. Low-satisfaction physicians however may be confused by these differing patient-information retrieval approaches, and thus regard these attributes of information quality to be overdeveloped.

As for service quality attributes, low- and high-satisfaction physicians in general consider the factor to be of less importance when compared with system quality and information quality since physicians are professionals who may exhibit considerable competence in adapting to new technologies [44]. Such a competence in using an HIS can also explain why SEQ4 was identified in the “Possible overkill” quadrant by high-satisfaction physicians. SEQ1 (“insufficient support from IT department”) was however identified in the “Concentrate here” quadrant by low-satisfaction physicians. This finding may indicate less-experienced physicians still wanting to focus on their patient-caring jobs and not wanting to be interrupted by any HIS problems during their immediate care of patients.

## 5. Conclusions

Due to the rapid progress of information and communication technologies, developing and adopting an HIS has been one of the critical means of improving health-care quality for many health-care facilities. There are numerous studies focusing on the factors influencing physicians' use of HIS from a varietal viewpoint of theories or models; however, exploring physicians' behavior from the perspective of the differences between importance and performance is rarely discussed. Our study conducted an empirical investigation among physicians utilizing the IPA method in order to provide a successful attempt of explanation relevant to attributes.

Using IPA, our study has compared the importance and performance of factors contributing to HIS satisfaction, as perceived by low- and high-satisfaction users. The IPA grids have demonstrated that system quality and service quality fell into the “Concentrate here” quadrant and information quality into the “Possible overkill” quadrant from the perspective of high-satisfaction HIS users. As for low-satisfaction HIS users, system quality was found in the “Concentrate here” quadrant, service quality in the “Low priority” quadrant, and information quality in the “Keep up the good work” quadrant. Improving system quality of respective HIS is considered to be the most important integer toward evincing physician users' satisfaction.

Via the use of IPA to explore the differences between the low- and high-satisfaction groups of HIS, physician users could add significant knowledge to further research studies in the area of IS success theory. Analyzing perceptions of quality in terms of differing segments can help hospitals to formulate developmental strategies of HIS to fulfill the demands of each specific segment. Based on these given findings, several implications can be derived. First, the health-care system may consider how to equip physicians with sufficient knowledge regarding the functions and information-retrieval approaches provided by their governing HIS. This implication is especially important for junior physicians; qualitative training sessions are thus a possible and a viable solution toward resolving this issue. Second, the IT department should provide sufficient support for physicians, especially for junior physicians interfacing the HIS, since patient care is the primary duty of physicians. If physicians can focus on patient care without other nonproductive interruptions, such as encountering HIS-usage problems, health-care quality can be substantially improved.

Some limitations should be noted in our study. First, our results are based on an HIS as implemented in a single health-care system in southern Taiwan; as a result, the generalizability may be somewhat limited. Furthermore, this study was conducted in a cross-sectional setting and might be unable to capture the varying perspectives of physicians over a period of time. Further efforts are thus necessary for the proposed model to be further implemented if longitudinal studies are to be conducted in the future.

## Figures and Tables

**Table 1:** Operational definitions of constructs investigated.

Constructs	Operational definition	References
System quality	Referring the desired technical characteristics such as the reliability, response time, and functionality of an HIS	[9, 16]
Information quality	Measuring the characteristics of the information output derived, such as sufficient detail, easy-to-read perception, and the completeness offered by an HIS	
Service quality	Relating to the level of support, such as availability, responsiveness, and training opportunities, of physician users by the IS department	
Satisfaction	Referring to the affective response or attitude of physician users toward an HIS	

**Table 2:** The final questionnaire of this study.

Constructs	Short name	Items	References
System quality (SQ)	SQ1	Our HIS performs reliably for my patient-care work	Balaban et al. [10]; Xu et al. [33]
	SQ2	The responsible time of our HIS is quick	
	SQ3	Our HIS provides necessary features and functions for my work	

Information quality (IQ)	IQ1	I can query patient information that I need from our HIS	Bossen et al. [11]; Xu et al. [33]
	IQ2	The information provided by our HIS is sufficiently detailed	
	IQ3	The information provided by our HIS is easy to read	

Service quality (SEQ)	SEQ1	The service provided by IT departments for HIS is sufficient	Balaban et al. [10]; Wang [15]
	SEQ2	Our IT department is available for assistance with difficulties when using a HIS	
	SEQ3	The training for HIS usage is sufficient in our hospital	
	SEQ4	When encountering problems in using a HIS, I can also find someone to help me	

Satisfaction (SAT)	SAT1	I am satisfied with our HIS	Cohen et al. [12]
	SAT2	I am pleased with using our HIS	
	SAT3	I found it enjoyable to use our HIS	
	SAT4	I have a favorable experience of using our HIS	
	SAT5	I have a positive attitude toward using our HIS for clinical care	

**Table 3:** Demographic information of respondents.

Attributes	Item	Full (*n*=150)		Low satisfaction (*n*=69)		High satisfaction (*n*=81)	
		Frequency	%	Frequency	%	Frequency	%
Gender	Male	119	79.33	58	38.76	61	40.67
	Female	31	20.67	11	7.33	20	13.33

Age (years)	20–29	19	12.67	8	5.33	11	7.33
	30–39	90	60.00	45	30.00	45	30.00
	40–49	32	21.33	15	10.00	17	11.33
	≥50	9	6.00	1	0.67	8	5.33

Job	Attending	102	68.00	47	31.33	55	36.67
	Resident	48	32.00	22	14.67	26	17.33

Speciality	General medicine	33	22.00	14	11.29	19	14.50
	General surgery	45	38.46	19	17.59	26	22.81
	Obstetrics and gynecology	12	10.34	3	2.70	9	8.18
	Pediatrics	11	9.24	6	5.22	5	4.59
	Emergency	14	10.00	10	8.20	4	3.25
	Others	35	31.25	17	15.45	18	16.82

Working experiences (years)	<1	7	4.67	5	3.33	2	1.33
	1–3	26	17.33	10	6.67	16	10.67
	4–6	37	24.67	17	11.33	20	13.33
	7–9	20	13.33	10	6.67	10	6.67
	≧10	60	40.00	27	18.00	33	22.00

HIS usage frequency (times per week)	1	6	4.00	3	2.00	3	2.00
	2–3	3	2.00	1	0.67	2	1.33
	4–6	45	30.00	22	14.67	23	15.33
	≧7	96	64.00	43	28.67	53	35.33

*Note.* Some numbers in this report may not add up due to rounding effect.

**Table 4:** Exploratory factor analysis.

Construct	Items	SAT	IQ	SQ	SEQ
System quality (SQ)	SQ1	0.19	−0.09	**0.74**	0.06
	SQ2	0.02	−0.01	**0.86**	0.04
	SQ3	0.24	0.40	**0.49**	−0.10

Information quality (IQ)	IQ1	−0.06	**0.92**	−0.04	0.06
	IQ2	0.10	**0.86**	0.03	−0.01
	IQ3	0.27	**0.69**	−0.03	0.05

Service quality (SEQ)	SEQ1	0.34	0.01	0.11	**0.56**
	SEQ2	−0.18	0.32	0.33	**0.61**
	SEQ3	0.18	−0.02	−0.09	**0.84**
	SEQ4	0.08	0.11	0.27	**0.56**

Satisfaction (SAT)	SAT1	**0.86**	0.06	0.01	0.05
	SAT2	**0.60**	0.23	0.19	−0.03
	SAT3	**0.89**	0.00	0.03	0.04
	SAT4	**0.90**	0.02	0.07	0.01
	SAT5	**0.72**	0.08	0.02	0.17

Eigenvalue		4.46	3.00	2.40	2.36
Variance explained (%)		29.73	20.03	16.01	15.74
Cronbach's *α*		0.95	0.89	0.83	0.90

**Table 5:** Reliability and validity.

Constructs	Items	Loadings	CR	AVE
		Full sample/low satisfaction/high satisfaction		
System quality (SQ) [10, 33]	SQ1	0.86/0.83/0.81	0.90/0.87/0.85	0.75/0.69/0.65
	SQ2	0.85/0.80/0.78		
	SQ3	0.89/0.87/0.83		

Information quality (IQ) [11, 33]	IQ1	0.87/0.74/0.90	0.93/0.89/0.94	0.83/0.73/0.84
	IQ2	0.94/0.91/0.93		
	IQ3	0.92/0.90/0.92		

Service quality (SEQ) [10, 15]	SEQ1	0.89/0.80/0.89	0.93/0.87/0.92	0.76/0.63/0.75
	SEQ2	0.90/0.84/0.91		
	SEQ3	0.83/0.74/0.77		
	SEQ4	0.88/0.78/0.89		

Satisfaction (SAT) [12]	SAT1	0.93/0.83/0.80	0.96/0.90/0.75	0.84/0.64/0.74
	SAT2	0.88/0.65/0.85		
	SAT3	0.93/0.85/0.87		
	SAT4	0.96/0.89/0.93		
	SAT5	0.89/0.77/0.85		

*Note.* CR denotes composite reliability; AVE denotes average variance extracted.

**Table 6:** Correlations among investigated constructs.

	*M*	SD	SAT	IQ	SQ	SEQ
Satisfaction (SAT)	4.19	1.15	**0.87**	—	—	—
Information quality (IQ)	4.80	1.08	0.67	**0.91**	—	—
System quality (SQ)	4.13	1.15	0.75	0.69	**0.87**	—
Service quality (SEQ)	4.07	1.27	0.79	0.73	0.78	**0.92**

*Note. M* means mean; SD denotes standard deviation.

**Table 7:** Summary of path coefficients and confidence interval.

Paths (→satisfaction)	Full sample/low satisfaction/high satisfaction		
	Path coefficient	*t*-statistics	95% BCa CI
System quality	0.37/0.38/0.39	4.08/2.51/4.99	[0.11, 0.51]/[0.01, 0.58]/[0.25, 0.53]
Information quality	0.26/0.19/0.38	3.36/1.74/4.25	[0.08, 0.40]/[−0.05, 0.36]/[0.16, 0.54]
Service quality	0.32/0.31/0.18	3.44/2.48/1.62	[0.17, 0.66]/[0.11, 0.60]/[−0.07, 0.38]

*Note.* BCa CI = bias-corrected and accelerated bootstrap confidence interval.

**Table 8:** Construct-level importance and performance index.

Sample	Construct	Performance			Importance index
		Index	Rank	Mean	
Full	System quality	53.39	2	56.17	0.37
	Information quality	63.03	1		0.26
	Service quality	52.08	3		0.32

Low-satisfaction	System quality	41.69	2	44.33	0.38
	Information quality	51.34	1		0.19
	Service quality	39.96	3		0.31

High-satisfaction	System quality	63.55	2	66.04	0.39
	Information quality	72.21	1		0.38
	Service quality	62.38	3		0.18

**Table 9:** Indicator-level importance and performance index.

Sample	Construct	Indicator	Performance	Mean	Importance
Full	System quality (SQ)	SQ1	52.44	55.81	0.14
		SQ2	50.67		0.13
		SQ3	56.44		0.16
	Information quality (IQ)	IQ1	68.00		0.08
		IQ2	61.67		0.10
		IQ3	60.33		0.10
	Service quality (SEQ)	SEQ1	47.78		0.10
		SEQ2	55.33		0.09
		SEQ3	50.22		0.08
		SEQ4	55.22		0.09

Low-satisfaction	System quality (SQ)	SQ1	41.79	44.44	0.16
		SQ2	39.61		0.13
		SQ3	43.24		0.16
	Information quality (IQ)	IQ1	60.14		0.04
		IQ2	50.97		0.08
		IQ3	47.58		0.09
	Service quality (SEQ)	SEQ1	34.06		0.12
		SEQ2	44.69		0.09
		SEQ3	39.61		0.08
		SEQ4	42.75		0.10

High-satisfaction	System quality (SQ)	SQ1	61.52	65.49	0.16
		SQ2	60.08		0.13
		SQ3	67.70		0.19
	Information quality (IQ)	IQ1	74.69		0.14
		IQ2	70.78		0.15
		IQ3	71.19		0.13
	Service quality (SEQ)	SEQ1	59.47		0.06
		SEQ2	64.40		0.05
		SEQ3	59.26		0.04
		SEQ4	65.84		0.06

## Data Availability

The data used to support the findings of this study are available from the corresponding author upon request.

## Abbreviations

AVE: Average variance extraction
BCa: Bias-corrected and accelerated bootstrap
CI: Confidence interval
CPOE: Computerized physician order entry
CR: Composite reliability
f2: Strength of each predictor variable in explaining endogenous variables
HIS: Hospital information systems
IPA: Importance-performance analysis
IQ: Information quality
ISSM: Information system success model
KMO: Kaiser-Meyer-Olkin
*M*: Mean
*p*: probability values
PLS: Partial least squares
*Q*^2^: Predictive relevance
*q*^2^: Relative impact of predictive relevance
*R*^2^: Coefficient of determination
SAT: Satisfaction
SD: Standard deviation
SEQ: Service quality
SQ: System quality
*t*: *t* statistics
*β*: Path coefficients.
